# INFORMATION SHARING TO SUPPORT CARE TRANSITIONS FOR PATIENTS WITH COMPLEX MENTAL AND BEHAVIORAL HEALTH NEEDS

**DOI:** 10.1093/geroni/igac059.997

**Published:** 2022-12-20

**Authors:** Taylor Bucy, Dori Cross

**Affiliations:** University of Minnesota School of Public Health, Minneapolis, Minnesota, United States; University of Minnesota School of Public Health, Minneapolis, Minnesota, United States

## Abstract

Information sharing practices between hospitals and skilled nursing facilities (SNFs) are insufficient to effectively support patient handoffs. Information needs are even greater for SNFs that admit patients with complex behavioral needs. It is unclear whether these needs have prompted hospital investment in enhanced information sharing with these SNFs, and what strategies these facilities are using to meet informational needs. We use data from a 2019 nationally representative SNF survey (N=265, response rate 53%) designed to gather information on information sharing practices with hospital partners. 122 SNFs (57% of respondents) report accepting at least two of the following complex conditions: serious mental illness, substance use disorder, or medication assisted treatment. Using logistic regression models that adjust for facility ownership and rurality, SNFs that accept complex patients are significantly more likely to receive information on behavioral, mental, and functional status compared to facilities who accept none or only one type of complex patient (odds ratio=2.42; p=0.023). Unadjusted models indicate that facilities that accept complex patients lag in IT-facilitated access to hospital information, and report more difficulty securing timely access to information. The significance of these findings do not persist after adjustment, suggesting structural differences in the types of SNFs that hospitals are partnering with to improve information sharing. We conclude that while SNFs that accept complex patients are mostly keeping pace or even doing slightly better in terms of access to hospital information that supports transitional care, further investment is needed to improve hospital information sharing behaviors.

